# Antibacterial effects of human mesenchymal stem cells and their derivatives: a systematic review

**DOI:** 10.3389/fmicb.2024.1430650

**Published:** 2024-09-25

**Authors:** Adeline Castro Ramos, Markus Yovian Widjaja Lomanto, Cat-Khanh Vuong, Osamu Ohneda, Mizuho Fukushige

**Affiliations:** ^1^Graduate School of Comprehensive Human Science, Laboratory of Regenerative Medicine and Stem Cell Biology, University of Tsukuba, Tsukuba, Japan; ^2^School of Integrative and Global Majors, Laboratory of Regenerative Medicine and Stem Cell Biology, University of Tsukuba, Tsukuba, Japan; ^3^Laboratory of Regenerative Medicine and Stem Cell Biology, Institute of Medicine, University of Tsukuba, Tsukuba, Japan; ^4^Department of Internal Medicine, National Cheng Kung University Hospital, College of Medicine, National Cheng Kung University, Tainan, Taiwan

**Keywords:** mesenchymal stem cells, mesenchymal stromal cells, antimicrobial resistance, antibacterial property, antibacterial activity

## Abstract

**Introduction:**

The growing problem of antimicrobial resistance (AMR) poses a significant challenge to public health; This is partly due to the lack of advancements in the development of novel antibiotics and the pressing need for alternative treatment options. Mesenchymal stem cells (MSC) possess secretory components that enhance the immune response and peptides that disrupt the bacteria constitution. The isolation of various human tissues has facilitated the investigation of the diverse potentials of MSC and their components. Further research is needed to fully understand the spectrum and efficacy of these elements and their differences. The primary aim of this study was to perform a thorough review of the current literature related to the antimicrobial properties of MSC and their associated components. The objective was to establish an insight into the results and effects of utilizing MSC in relation to bacterial colonization, and to present an overview of previously documented findings.

**Methods:**

This systematic literature review was conducted using the PubMed, Embase, and Web of Science databases. Data on the effect of MSC or their derivatives were measured by calculating the percentage of bacterial counts reduction after treatment with MSC in comparison to the control.

**Results:**

A total of 3,911 articles were screened, and 31 eligible publications were selected for inclusion in the analysis. In the current systematic review, the majority of the experimental designs showed positive outcomes in terms of bacterial load reduction when MSC or their derivatives were used, with bone marrow being the most effective tissue. The rest of the findings exhibited heterogeneity in the spectrum of outcomes that could be attributed to the effects of using various tissues derived MSC in both *in vivo* and *in vitro* studies.

**Conclusion:**

The findings of our study indicate the potential antibacterial characteristics of MSC. The direct antimicrobial activity of these cells was demonstrated by our results, which quantitatively showed a decrease in bacterial growth after treatment with MSC. However, additional research is required to clarify the factors that determine the efficacy of their antimicrobial activity and their various components.

## 1 Introduction

Antimicrobial resistance (AMR) is a state in which pathogens acquire DNA-driven mechanisms to resist antibiotics. As more microorganisms acquire these modifications, treating infections becomes increasingly challenging (Centers for Disease Control and Prevention, 2022). When a pathogen cannot be controlled, it represents a risk to public health. The ongoing global issue of AMR has cost 1,170,000 lives (Centers for Disease Control and Prevention, 2022). Without novel treatment approaches, healthcare procedures such as surgery may become difficult in the near future (World Health Organization, 2023). Another factor contributing to the intensification of this issue is the stagnation of new antibiotic development. AMR is a complicated public health problem because it lengthens hospital stays, intensifies care for patients with infectious diseases, raises healthcare expenses, and spreads through humans, animals, and food supplies (World Health Organization, 2023).

Mesenchymal stem cells (MSC) are a prospect in the search for new therapeutic approaches. In addition to their known regenerative properties. They have been shown to have antibacterial action (Alcayaga-Miranda et al., 2017). These effects are primarily mediated by the immunoregulation they exert on phagocytic activity and the secretion of products with bactericidal effects, such as antibacterial peptides (Alcayaga-Miranda et al., 2017). These have previously been reported to have pharmacodynamic curves that are distinct from those of antibiotics and are less susceptible to the development of resistance (El Shazely et al., 2020). Additionally, it has been described as having an elevated selectivity index, displaying a specific affinity to the bacterium, a property that could confer less toxicity on host cells (Bakare et al., 2023), with this mode of action being the defining property that renders MSC an alternative to current therapeutics. Numerous peptide families, including cathelicidins, defensins, hepcidins, and lipocalins, have been identified in MSC (Alcayaga-Miranda et al., 2017). The antibacterial effect of MSC has also been described to have various ranges of action (Alcayaga-Miranda et al., 2017). Previous studies have shown that MSC are capable of inhibiting the growth of both Gram-positive and Gram-negative bacteria, as well as some multidrug-resistant strains (Bakare et al., 2023).

Although a broad spectrum of antibacterial role has been described previously, it is important to note that several factors can modify the antibacterial potential of MSC. Environmental factors, such as the interaction with other cells, the different potential of MSCs from various tissues (Bakare et al., 2023) and the route of administration (Shaw et al., 2021) may influence their antibacterial capacity. Numerous studies have focused on the use of MSC-derived soluble factors and has been previously described as having anti-inflammatory and immunomodulatory properties (Műzes and Sipos, 2022). Future experimental research should, characterize and describe these factors, determine the variabilities associated with them, especially those related to the tissue source of MSC and the antibacterial capacity of their derived components is particularly important because knowing the advantages of one source over another enables us to understand the favorable mechanisms of each type of MSC. Therefore, it is important to conduct comparative studies of MSC, especially to describe their antimicrobial properties, considering that characterizing these properties could elucidate potential antibacterial applications of MSC.

Hence, the primary objective of this study was to conduct a thorough review of the existing literature on the antimicrobial capabilities of MSC and their derived factors. The aim was to identify gaps in the current knowledge, ascertain the outcomes and impacts of utilizing different types of MSC, describe

and compare the effects achieved by their use in various tissues, and provide a comprehensive overview of previously reported findings. This analysis will serve as a foundation for future preclinical investigations and for exploring the potential role of MSC in addressing bacterial infections.

## 2 Materials and methods

### 2.1 Search strategy

In the present systematic review, a search for potential articles was conducted using the electronic databases PubMed, Embase, and Web of Science. The search was completed on March 4, 2023. Year restrictions were not imposed in the search strategy. The search terms used were (antibiotic OR antibacterial) AND mesenchymal stem cells. Original articles, including those relevant to our research purpose, were extracted after manual screening of systematic reviews. The eligibility of the articles was screened by two independent reviewers: ACR and MWL.

### 2.2 Eligibility criteria for the systematic reviews

The reference manager EndNote version 20.2.1 and Hubmeta (hubmeta.com) were used to remove duplicates. Relevant articles were selected based on the title and abstract. Subsequently, an in-depth selection was conducted according to the inclusion criteria of the full articles, which finally identified 31 articles that were included in the present review.

Studies were eligible for inclusion if they met all of the following criteria: 1) used microvesicles (MVs), extracellular vesicles (EVs), conditioned medium (CM), or supernatant derived from MSC; 2) reported bacterial ingrowth or outgrowth in terms of CFU or OD absorbance; 3) used MSC from any human tissue source; and 4) used *in vivo* or *in vitro* models. The exclusion criteria were: 1) written in any language other than English or Spanish; 2) use of hydrogels, scaffolds, nanoparticles, or any other biomaterial; 3) use of MSC as an adjuvant for antibiotic therapy; 4) use of MSC as a co-factor for another main treatment (immune cells, signal factors, cytokines); and 5) published as posters, conference papers, letters to the editor, or presentations.

### 2.3 Data extraction

The findings regarding the experimental design, MSC and bacteria employed, type of infection studied, and results related to bacterial growth was extracted from the selected articles. The percentage of reduction in bacterial numbers after treatment with MSC or derivatives on bacterial growth was determined after the extraction of the mean of the figures from the experimental data using the Webplot Digitizer software program (version 4.6)^1^ and the descriptive text of the article results. Experimental results from articles presenting data expressed as medians, percentiles, or units other than CFU were not extracted for the quantitative analysis. To determine the percentage reduction in total bacterial count, the difference between the mean of the number of bacterial counts of the control (A) and the mean of the number of bacterial counts of the MSC treatment groups (B) in relation to the mean of the control (A) was calculated using the following formula:

Percentage of bacteria reduction was calculated using the following formula:


$$\%=\frac{M\,e\,a\,n\,o\,f\,\left(A\right)-M\,e\,a\,n\,o\,f\,\left(B\right)}{M\,e\,a\,n\,o\,f\,\left(A\right)}\times 100$$

## 3 Results

### 3.1 Characteristics of included studies

**FIGURE 1:**
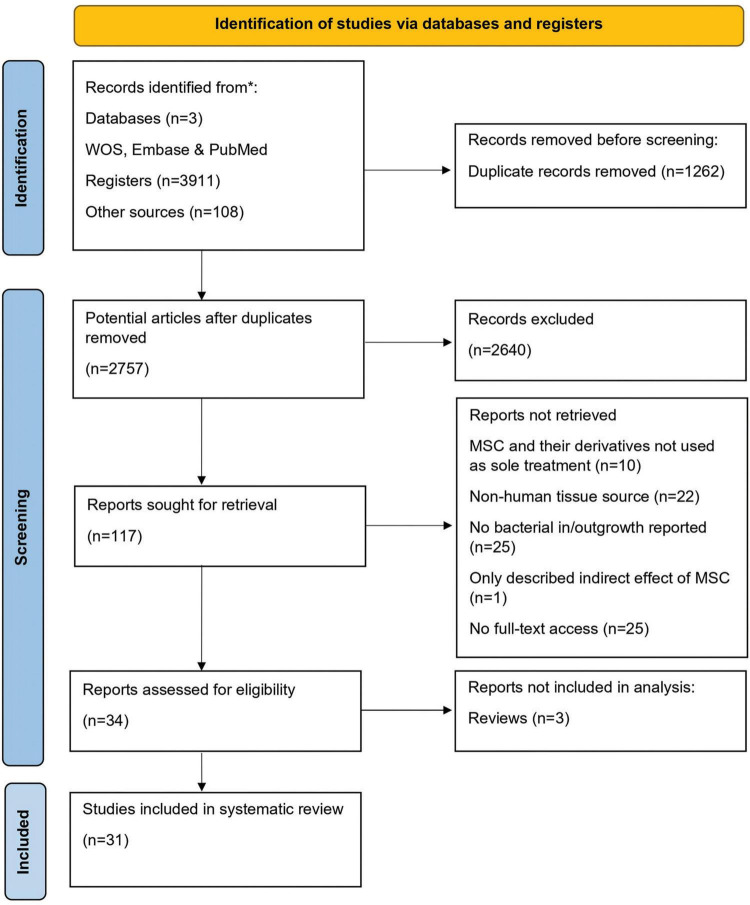
PRISMA Flow diagram. A systematic review flow diagram representing the number of articles identified and examined at each stage of the review. A total of 31 articles were included for the systematic review. Adapted from PRISMA website (http://prisma-statement.org/prismastatement/flowdiagram.asp).

A systematic search of electronic databases yielded 3,911 articles. In addition, 108 articles were identified from five review papers. After excluding 1,262 duplicates, 2,757 articles were obtained. Further screening of titles and abstracts led to the exclusion of an additional 2,640 articles. Full-text assessment was conducted on the remaining 116 articles. Among these, 83 did not meet the inclusion criteria, resulting in a final selection of 34 articles for the current systematic review, three of them being reviews. Finally, 31 articles were used for data extraction and the analysis of the antibacterial effects of MSC (Ahn et al., 2018; Asmussen et al., 2014; Bahroudi et al., 2020; Bonfield et al., 2021; Chow et al., 2020; Devaney et al., 2015; Dubus et al., 2020; Gonzalez et al., 2020; Horie et al., 2020; Kim et al., 2022; Krasnodembskaya et al., 2012, 2010; Laroye et al., 2018; Lee et al., 2013; Li et al., 2020; Masterson et al., 2018; McCarthy et al., 2020; Monsarrat et al., 2019; Monsel et al., 2015; Park et al., 2019; Perlee et al., 2019; Ravenscroft et al., 2022; Ren et al., 2020; Sung et al., 2016; Sutton et al., 2017; Varkouhi et al., 2021; Wang et al., 2023; Wood et al., 2018; Yagi et al., 2020; Yang et al., 2022; Zhu et al., 2017; Figure 1 and Table 1).

**TABLE 1:** Summary of selected studies.

Appendix reference	Year	Author	Type of Experiment	Pathogen	Type of Tissue-MSC	Animal Model	Use	Type of Infection	Antibacterial Outcomes
1	2020	Bahroudi et al.	*In vitro*	*V. cholerae*	Bone Marrow	-	Supernatant	Chronic Infection	⇊CFU: Significantly decreased bacterial suspension, antibiofilm activity
2	2010	Krasnodembs kaya et al.	*In vitro In vivo*	*E. coli, P. aeruginosa, and S. aureus*	Bone Marrow	C57BL/ 6 male mice	Cell and conditioned medium	Respiratory	⇊CFU: *In vitro*: CM inhibited *E. coli* and *P. aeruginosa*. MSC significantly inhibit *E. coli* and *S. aureus* growth. *In vivo:* MSC reduced *E. coli* growth. Antibacterial factor: LL-37
3	2015	Sung et al.	*In vitro In vivo*	*E. coli*	Umbilical Cord	ICR mice	Cell and conditioned medium	Respiratory	⇊CFU: *In vitro:* MSC significantly inhibited bacterial growth, CM (precondition through the interaction with the bacteria) significantly inhibited bacterial growth, *In vivo*: Antibacterial effect of MSC in BAL infected with*E. coli*, effect was abolished with TLR-4 stimulation siRNA. Antibacterial factor: β- defensin-2
4	2020	Yagi et al.	*In vitro*	*S. aureus*	Adipose (Infrapatellar) and Bone Marrow (Femoral bone)	–	Cell and conditioned medium	Bone Infection	⇊CFU: CM alone from BM and AT derived MSC significantly inhibited bacterial growth. BM and AT MSC significantly inhibited bacterial growth in synovial fluid co cultured with *S. aureus.* Antibacterial factor: LL-37
5	2013	Lee et al.	*Ex vivo perfused human lung In vitro*	*E. coli*	Bone Marrow	Ex vivo human lung	Cell	Respiratory	⇊CFU: MSC significantly reduced the bacterial load, Alveolar fluid from E. coli- exposed lungs treated with MSCs showed enhanced antimicrobial activity. Bacteremia was reduced with MSC treatment. Antibacterial factor: KGF
6	2019	Monsarrat et al.	*In vitro In vivo*	*F. nucleatum, P. gingivalis, P. intermedia, S. sanguinis, E. faecalis, L. casei, S.aureus, E. coli and Wild peripatogenic bacteria.*	Adipose (Subcutane us)	Murine model of periodo ntitis	Cell	Dental Infection	⇊CFU: *In vivo:* MSC significantly decreased the number of CFUs formation in subgingival murine model.*In vitro*: ⇊ Reduce Kineticgrowth: Significant decreasein the growth rate whentreated with AT- MSC, aswell a significantly decreased the CFU number of four (*E.coli, S. aureus, S. sanguinisand L. casei*) of eight strains was reported.
7	2021	Bonfield et al.	*In vitro In vivo*	*Non tuberculous mycobacteria M. avium, M. intracellulare and Mycobacterium avium complex*	Bone Marrow	C57BL/6J; *Cftr* tm1 Kth Tg1 Jaw/Cwr and Cftr tm1Kth	Cell and supernatant	Respiratory	⇊CFU: *In vitro*: MSC significantly decreased bacterial CFU counts. Supernatant significantly decrease MAC (*Mycobacterium avium complex*) only. *In vivo*: Treatment with MSC decreased CFUs from *M. intracellulare* and *M. avium*.
8	2019	Perlee et al.	*In vivo*	*K. pneumoniae serotype 2*	Adipose	C57BL/ 6 mice	Cell (culture and cryopreserved)	Respiratory	⇊CFU: Cryopreserve decreased bacterial load at 16hrs post infection in lungs and not distant organs. Culture MSC showed antibacterial effect in lungs and distant organs at 48hrs after infusion.
9	2018	Wood et al.	*In vitro*	*S. aureus and P. aeruginosa*	Adipose	–	Conditioned medium	Chronic infection	⇊CFU: CM precondition with the interaction with bacteria inhibit both bacteria’s growth. CM non primed mainly decreased *S. aureus*. Antibiofilm activity was also reported to the use of conditioned medium.
10	2020	Ren et al.	*In vitro*	*IRPA*	Umbilical Cord	–	Cells and conditioned medium	Neonatal infection	⇊CFU: CM stimulated with *P. aeruginosa* and UC derived MSC decreased bacterial growth. Antibacterial factor: LL-37 and β-defensin-2
11	2022	Yang et al.	*In vitro*	*P. aeruginosa*	Umbilical cord	–	Supernatant	Chronic Infection	⇊Bacterial growth: Supernatant decrease the biofilm formation in artificial tracheal tube. Antibacterial factor: LL-37 and β-defensin-2
12	2020	Dubus et al.	*In vitro*	*C. acnes and S. aureus*	Bone Marrow (Femoral neck)	–	Conditioned medium	Implanted associated Infection	⇊Bacterial Growth: 25% decreased on 2 of 3 strains of *C. acnes* infection with the use of CM. Antibacterial activity was not enhanced by CM previously infected *C. acnes*. No bactericidal effect to C2 strain. Antibiofilm effect was seen after used of CM on one *S. aureus* strain.
13	2022	Kim et al.	*In vivo In vitro*	*E. coli K1 capsular polysaccharide*	Umbilical cord (Wharton Jelly)	Sprague– Dawley	Extracellular vesicle and cells	Brain Infection	⇊CFU. *In vitro*: MSC effective bacterial clearance in the culture media. ↓CFU. *In vivo:* EVs didn’t significantly had an effect.
14	2018	Ahn et al.	*In vivo*	*E. coli K1 capsular polysaccharide C5*	Umbilical cord	Sprague– Dawley	Cells	Brain Infection	⇊CFU. Decreased of bacterial count on the study groups treated with MSCs.
15	2020	McCarthy et al.	*In vitro*	*E. coli, S. aureus and K. pneumoniae*	Umbilical cord and Bone Marrow	–	Conditioned medium	Respiratory	⇊ Bacterial growth: The use of nebulized CM from BM- MSCs reduced *E. coli, K. pneumoniae and S. aureus.* UC derived MSC showed same effect reducing OD600 of the three bacteria strains.
16	2022	Ravenscroft, et al.	*In vitro*	*S. aureus, E.coli, S. mutans, L. acidophilus and F. nucleatum.*	Dental Pulp	–	Conditioned medium	Dental infection	⇊CFU. The number of CFU of all bacteria strain tested were significantly reduced. Antibacterial factors: ANGPT-1, HGF, IL-8, and IL-6.
17	2023	Wang et al.	*In vivo In vitro*	*K. pneumoniae*	Placenta	C57BL/6	Cells	Respiratory	×CFU. *In vitro*: The growth of *K. Pneumoniae* was not affected by the used of Placenta derived MSCs. *In vivo:* MSC decreased bacterial load on lungs homogenates.
18	2020	Gonzalez et al.	*In vivo In vitro*	*Polymicrobial sepsis*	Umbilical cord	Sprague Dawley rats	Conditioned medium and cell	Abdominal infection	⇊CFU. *In vitro*: CM of MSC increased phagocytosis in THP-1- derived Macrophages. *In vivo:* Bacterial genome copies were reduced after treatment with CD362+ UC derived MSC. As well, treatment showed bacterial clearance in liver and spleen where was reported a decreased CFU counts and an increase of antimicrobial peptides concentration. Antibacterial factor: Hepcidin
19	2020	Horie et al.	*In vivo*	*E. coli*	Bone marrow, Umbilical cord and Umbilical cord CD362+	Sprague Dawley rats	Cell	Respiratory	⇊CFU. Bacterial count was significantly decreased in the BAL in all treated groups with the three MSCs. There as a high and significant antibacterial effect.
20	2015	Monsel et al.	*In vivo*	*E. coli K1*	Bone Marrow	C57BL/ 6 mice	Micro vesicles and cells	Respiratory	⇊CFU. Intravenous administration of MVs significantly decrease the bacterial load in BAL, the intratracheal administration reduce 40% bacterial load in BAL but was not statistically significant. There was a significant decreased to the use of MSCs alone.
21	2012	Krasnodembs kaya et al.	*In vivo*	*P. aeruginosa*	Bone Marrow	C57BL/ 6J mice	Cell	Sepsis-General Infection	⇊CFU. Lower bacterial count was reported in peritoneal fluid, spleen and lungs but not significantly different to the control. It was found a significant decreased in bacterial count from blood.
22	2014	Asmussen et al.	*In vivo*	*P. aeruginosa*	Bone Marrow	Adult sheep	Cell	Respiratory	⇊CFU. To the comparison of control and treatment there was no differences in the median number of the low and high dose MSCs treated animals and the control.
23	2015	Devaney et al	*In vivo*	*E. coli*	Bone Marrow	Sprague Dawley rats	Cell and conditioned medium	Respiratory	⇊CFU. Doses above 10million MSCs/ kg reduced bacterial load. Intravenous and intratracheal administration showed a significant decreased of bacterial number. ×CFU. The CM of MSCs didn’t significantly reduce bacterial load. Antibacterial factor: LL-37
24	2017	Sutton et al.	*In vivo In vitro*	*P. aeruginosa and S. aureus*	Bone Marrow	*_*Cftr*_tm1Kth* (CF) and C57BL/ 6 (WT) mice	Cell	Respiratory	⇊CFU. The bacterial count from *P. aeruginosa* and *S. aureus* was significantly decreased with the MSC treatment in both *Cftr^tm1kth^* (CF) and C57BL/6 (WT) mice models. Antibacterial factor: LL-37 and CCL20
25	2018	Laroye et al.	*In vivo In vitro*	*P. aeruginosa, B. fragilis, and S. aureus*	Umbilical cord	Domestic pigs	Cell	Abdominal infection	×CFU. *In vivo*: CFU counts from control in comparison to MSC treated groups didn’t report significant differences in blood samples. *In vitro:* No antibacterial effect was observed on the three bacterial strains tested.
26	2018	Masterson et al.	*In vivo In vitro*	*E. coli*	Bone Marrow heterogeneous and CD362+	Sprague Dawley	Cell	Respiratory	⇊CFU. *In vivo:* CD362+ of MSC and heterogeneous MSC decreased *E. coli* counts. *In vitro*: Increased phagocytosis.
27	2019	Park et al.	Ex vivo	*E. coli*	Bone Marrow	Ex vivo lung human model	Microvesicles	Respiratory	⇊CFU. Administration of TLR-3 precondition MVs derived MSCs significantly decreased bacterial count. CFU counts were also lower but not significant in BAL after the administration in double doses of MVs precondition and MVs not previously conditioned.
28	2020	Chow et al.	*In vivo In vitro*	*MRSA and E. coli*	Bone Marrow	nu/nu mice	Conditioned medium	Acute and Chronic Infection	⇊CFU. *In vitro*: Bacterial count were significantly decreased in *MRSA* and *E. coli* treated with CM. The effect was obtained within the passage 1 to 9. Antibacterial factor: LL-37 and β-defensin- 2
29	2020	Li et al.	*In vivo*	*H. influenzae*	Umbilical cord	C57BL/ 6 mice	Cell	Respiratory	⇊ CFU. MSC inhibited bacterial proliferation in the lung and BAL
30	2021	Varkouhi et al.	*In vivo*	*Polymicrobial sepsis*	Umbilical cord and Bone Marrow	C57BL/ 6 male mice	Cell	Sepsis- General Infection	⇊ CFU. Only BM derived MSC significantly decreased bacterial load in blood, lungs and spleen tissue. ×CFU. There was no antibacterial effect reported to the use of UC- MSCs.
31	2017	Zhu et al.	*In vivo*	*E. coli serotype K1*	Umbilical cord	Sprague Dawley rats	Cell	Sepsis- General Infection	⇊ CFU. There was a significantly reduction of bacterial load in blood, lungs and spleen samples. Antibacterial factor: LL-37

Table shows the antibacterial effects of MSC, and their principal results with respect to experimental design and outcomes after interaction with bacteria. Antibacterial outcomes: ⇊ CFU, decreased in Colony Forming Units; ×CFU, fail to decrease Colony Forming Units; ⇊ Bacterial growth, decreased bacterial count in terms of OD absorbance. BAL, bronchoalveolar lavage; siRNA, short interfering RNA; MAC, Mycobacterium avium complex consisting of 50% *M. avium* and 10% *M. intracellulare*; *Cftr^tm1kth^*, Cystic fibrosis (CF) transmembrane receptor- deficient animals; C57BL/6, wild type (WT) mice; CM, conditioned medium; EVs, Extracellular vesicle; MVs, microvesicles; KGF, Keratinocyte Growth Factor; ANGPT-1, Angiopoietin-1; HGF, Hepatocyte Growth Factor; IL-8, Interleukin-8; IL-6, Interleukin-6.

**FIGURE 2:**
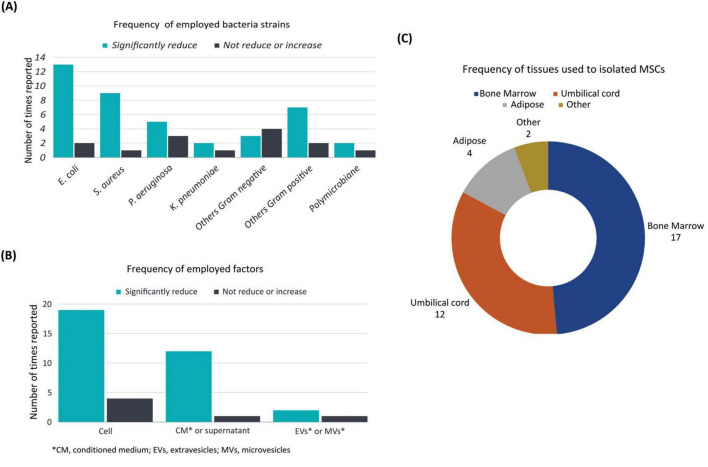
Frequency of the main characteristics. Main characteristics corresponding to the 31 articles analyzed showing the proportion of: **(A)** employed bacteria strains **(B)** employed MSC or their derived factors and **(C)** tissues used to isolate MSC in terms of number of times reported or included in the studies.

Among the reviewed articles, the antibacterial effects of MSC were studied using different bacteria (Table 1). Specifically, 15 studies reported the inclusion of *Escherichia coli*, while nine studies reported *Staphylococcus aureus*, thus establishing these microorganisms as the most commonly employed in the studies. *Pseudomonas aeruginosa* was reported in eight studies, while *Klebsiella pneumoniae* was reported in three studies. Furthermore, the use of other gram-negative bacteria, including *Fusobacterium nucleatum, Porphyromonas gingivalis, Prevotella intermedia, Bacteroides fragilis, Haemophilus influenzae* and *Vibrio cholerae* were reported in a total of seven studies. The use of gram-positive bacteria other than those previously mentioned was reported in ten studies, including the utilization of *Streptococcus sanguinis*, *Enterococcus faecalis*, *Lactobacillus casei*, *Mycobacterium avium, Mycobacterium intracellulare, Cutibacterium acnes, Streptococcus mutan*s, and *Lactobacillus acidophilus*. Notably, only two studies employed a polymicrobial infection model (Figure 2A and Table 1) and only two studies employed resistant strains utilizing *Methicillin-resistant Staphylococcus aureus* and *Imipenem-resistant Pseudomonas aeruginosa* (Table 1). The majority of studies reported a reduction in the bacterial count for each strain. However, in the case of Gram- negative bacteria (other than *P. aeruginosa, K. pneumoniae* or *E. coli*), most studies reported non- significant results or an increase in bacterial load (Figure 2A).

Based on the factors used to assess the efficacy of antibacterial treatment, MSC were used in 22 articles, while supernatant and conditioned medium derived from cellular excretion products were used in 13 articles. The use of MVs and EVs, however, was reported in only three articles (Figure 2B).

The antibacterial action of MSC was mostly attributed to the secretion of LL-37 peptide, which was consistently mentioned in eight studies (Krasnodembskaya et al., 2010; Yagi et al., 2020; Ren et al., 2020; Yang et al., 2022; Devaney et al., 2015; Sutton et al., 2017; Chow et al., 2020; Zhu et al., 2017). This peptide was closely associated with the antibacterial function of MSC. However, LL-37 was not the only component linked to the antibacterial effects of MSC. A total of four studies (Sung et al., 2016; Ren et al., 2020; Yang et al., 2022; Chow et al., 2020) identified β-defensin-2 as a MSC component involved in the bacterial inhibitory mechanism. One study (Lee et al., 2013) described KGF (Keratinocyte Growth Factor) was linked to the antibacterial mechanism of action. In addition, one study (Ravenscroft et al., 2022) indicated that several cytokines and growth factors, such as ANGPT- 1 (Angiopoietin-1), HGF (Hepatocyte Growth Factor), IL-8 (Interleukin-8), and IL-6 (Interleukin-6), were secreted and contributed to the antibacterial characteristics of MSC. Hepcidin was reported as an antibacterial agent in one study (Gonzalez et al., 2020). Only one study (Sutton et al., 2017) indicated

the MSC production of CCL20, a chemokine that possesses both chemotactic and antibacterial capabilities, (Table 1).

The present systematic review showed that bone marrow (BM)-derived MSC were the predominant selection in experimental designs, as evidenced by documentation of their utilization in 17 studies. Umbilical cord (UC)-derived MSC were the next most frequently used, appearing in 12 studies. A limited number of studies employed MSC isolated from adipose tissue (AT-MSC), with their application employed in a smaller subset of studies, appearing in only four articles. Cells obtained from the dental pulp (DP-MSC) and placenta (P-MSC) were utilized in only two studies (Figure 2C).

### 3.2 *In vitro* effects of MSC treatment against bacteria

**FIGURE 3:**
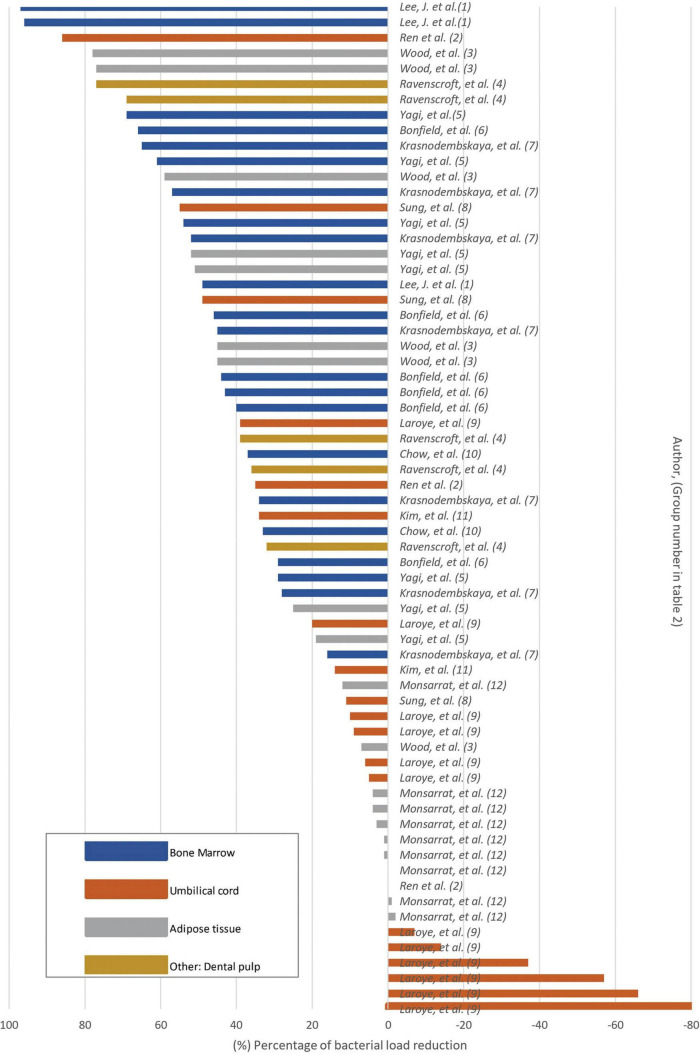
Bacterial load reduction *in vitro* Bar plot. Percentage of bacterial load reduction after MSC or MSC-derivative treatment in vitro. Horizontal bar plot showing percentage reduction after comparing MSCs or MSC-derived factors in comparison to control.

Twelve *in vitro* studies were identified among the selected studies. The findings of our systematic review showed that the majority of studies reported a significant reduction in bacterial count when MSC or their derivatives were used (Figure 3 and Table 2). A single study reported that treatment with UC-MSC at various concentrations resulted in bacterial proliferation (Laroye et al., 2018; Table 2). The remaining results exhibited a wide range, encompassing both non-reduction and significant reduction, with variations depending on the specific bacteria and MSC type or derivative employed (Table 2).

**TABLE 2:** Percentage reduction of bacterial load *in vitro*.

Group Number	Author – (Appendix reference)	Year	Bacteria	Antibacterial outcomes	Factor used	Parameter measured	%
1	Lee et al. (5)	2012	*E. coli*	Significantly reduced bacteria load	Alveolar fluid conditioned medium from lungs treated with BM MSC Intravenously doble dose	Bacterial load CFU/ml	97
	Lee et al. (5)	2012	*E. coli*	Significantly reduced bacteria load	Alveolar fluid conditioned medium from lungs treated with BM MSC Intrabronchially	Bacterial load CFU/ml	96
	Lee et al. (5)	2012	*E. coli*	Increased antimicrobial activity	Alveolar fluid conditioned medium from lungs treated with BM MSC Intravenously single dose	Bacterial load CFU/ml	49
2	Ren et al. (10)	2020	*IRPA*	Significantly reduced bacteria load	UC MSC stimulated with bacteria	Bacterial load CFU/ml	86
	Ren et al. (10)	2020	*IRPA*	Significantly reduced bacteria load	CM from UC MSC stimulated with bacteria	Bacterial load CFU/ml	35
	Ren et al. (10)	2020	*IRPA*	Not significantly reduced bacteria load	CM from UC MSC unstimulated with Bacteria	Bacterial load CFU/ml	0
3	Wood et al. (9)	2018	*S. aureus*	Significantly reduced bacteria load	CM from AT MSC primed with *S. aureus*	Bacterial load percentage of control	78
	Wood et al. (9)	2018	*P. aeruginosa*	Significantly reduced bacteria load	CM from AT MSC primed with *S. aureus*	Bacterial load percentage of control	77
	Wood et al. (9)	2018	*S. aureus*	Significantly reduced bacteria load	CM from AT MSC not primed	Bacterial load percentage of control	59
	Wood et al. (9)	2018	*S. aureus*	Significantly reduced bacteria load	CM from AT MSC primed with *P. aeruginosa*	Bacterial load percentage of control	45
	Wood et al. (9)	2018	*P. aeruginosa*	Significantly reduced bacteria load	CM from AT MSC primed with *P. aeruginosa*	Bacterial load percentage of control	45
	Wood et al.(9)	2018	*P. aeruginosa*	Not significantly reduced bacteria load	CM from AT MSC not primed	Bacterial load percentage of Control	7
4	Ravenscroft et al. (16)	2022	*S. aureus*	Significantly reduced bacteria load	CM from DP MSC	Bacterial load CFU/ml	77
	Ravenscroft et al. (16)	2022	*S. mutans*	Significantly reduced bacteria load	CM from DP MSC	Bacterial load CFU/ml	69
	Ravenscroft et al. (16)	2022	*L. acidophilus*	Significantly reduced bacteria load	CM from DP MSC	Bacterial load CFU/ml	39
	Ravenscroft et al. (16)	2022	*F. nucleatum*	Significantly reduced bacteria load	CM from DP MSC	Bacterial load CFU/ml	36
	Ravenscroft et al. (16)	2022	*E. coli*	Significantly reduced bacteria load	CM from DP MSC	Bacterial load CFU/ml	32
5	Yagi et al. (4)	2020	*S. aureus*	Significantly reduced bacteria load	CM from BM MSC	Bacterial load CFU percentage of control	69
	Yagi et al. (4)	2020	*S. aureus*	Significantly reduced bacteria load	CM with BM MSC	Bacterial load CFU percentage of control	61
	Yagi et al. (4)	2020	*S. aureus*	Not significantly reduced bacteria load	BM MSC	Bacterial load CFU percentage of control	54
	Yagi et al. (4)	2020	*S. aureus*	Significantly reduced bacteria load	CM from AT MSC	Bacterial load CFU percentage of control	52
	Yagi et al. (4)	2020	*S. aureus*	Significantly reduced bacteria load	CM with AT MSC	Bacterial load CFU percentage of control	51
	Yagi et al. (4)	2020	*S. aureus*	Significantly reduced bacteria load	BM MSC	Bacterial load growth in synovial fluid percentage of control	29
	Yagi et al. (4)	2020	*S. aureus*	Significantly reduced bacteria load	AT MSC	Bacterial load growth in synovial fluid percentage of control	25
	Yagi et al. (4)	2020	*S. aureus*	Not significantly Reduced bacteria load	AT MSC	Bacterial load CFU percentage of control	19
6	Bonfield et al. (7)	2021	*M. avium*	Significantly reduced bacterial load	BM MSC	Bacteria load CFU	66
	Bonfield et al. (7)	2021	*M. avium*	Not significantly reduced bacterial load	Supernatant from BM MSC	Bacteria load CFU	46
	Bonfield et al. (7)	2021	*M. avium complex*	Significantly reduced bacteria load	Supernatant from BM MSC	Bacteria load CFU	44
	Bonfield et al. (7)	2021	*M. intracellulare*	Reduced bacterial load but not significantly	Supernatant from BM MSC	Bacteria load CFU	43
	Bonfield et al. (7)	2021	*M. intracellulare*	Reduced bacterial load but not significantly	BM-MSC	Bacterial load CFU	40
	Bonfield et al. (7)	2021	*M. aviumcomplex*	Significantly reduced bacterial load	BM-MSC	Bacterial load CFU	29
7	Krasnodemb skaya et al. (2)	2010	*P. aeruginosa*	Significantly reduced bacterial load	Stimulated with *E. coli* CM derived from BM MSC	Bacterial load CFU/ml	65
	Krasnodemb skaya et al. (2)	2010	*E. coli*	Significantly reduced bacterial load	BM MSCs stimulated with *E. coli*	Bacterial load CFU/ml	57
	Krasnodemb skaya et al. (2)	2010	*S. aureus*	Significantly reduced bacterial load	BM-MSC	Bacterial load CFU/ml	52
	Krasnodemb skaya et al. (2)	2010	*S. aureus*	Significantly reduced bacterial load	BM MSC derived CM after *S. aureus* stimulation	Bacterial load CFU/ml	45
	Krasnodemb skaya et al. (2)	2010	*E. coli*	Significantly reduced bacterial load	BM MSC derived CM after *E. coli* stimulation	Bacterial load CFU/ml	34
	Krasnodemb skaya et al. (2)	2010	*P. aeruginosa*	Not significantly reduced bacterial load	Unstimulated CM derived BM- MSC	Bacterial load CFU/ml	28
	Krasnodemb skaya et al. (2)	2010	*E. coli*	Not significantly reduced bacterial load	Unstimulated CM derived BM- MSC	Bacterial load CFU/ml	16
8	Sung et al. (3)	2015	*E. coli*	Significantly reduced bacterial load	UC-MSC	Bacterial CFU load/ml	55
	Sung etal. (3)	2015	*E. coli*	Significantly inhibited	CM from UC MSC with	Bacterial CFU load/ml	49
				bacteria growth	bacteria preconditioning		
	Sung et al. (3)	2015	*E. coli*	Not significantly inhibited bacteria growth	CM from UC MSC without bacteria preconditioning	Bacterial CFU load/ml	11
9	Laroye et al. (25)	2018	*P. aeruginosa*	Not significantly decreased bacterial load	UC MSC 10^3 concentration	Bacterial CFU load/ml	39
	Laroye et al. (25)	2018	*P. aeruginosa*	Not significantly decreased bacterial load	UC MSC 10^5 concentration	Bacterial CFU load/ml	20
	Laroye et al. (25)	2018	*P. aeruginosa*	Not significantly decreased bacterial load	UC MSC 10^4 concentration	Bacterial CFU load/ml	10
	Laroye et al. (25)	2018	*B. Fragilis*	Not significantly decreased bacterial load	UC MSC 10^5 concentration	Bacterial CFU load/ml	9
	Laroye et al. (25)	2018	*B. Fragilis*	Not significantly decreased bacterial Load	UC MSC 10^4 concentration	Bacterial CFU load/ml	6
	Laroye et al. (25)	2018	*P. aeruginosa*	Not significantly decreased bacterial load	UC MSC 10^6 concentration	Bacterial CFU load/ml	5
	Laroye et al. (25)	2018	*B. Fragilis*	Not significantly decreased bacterial load	UC MSC 10^3 concentration	Bacterial CFU load/ml	−7
	Laroye et al. (25)	2018	*S. aureus*	Not significantly decreased bacterial load	UC MSC 10^4 concentration	Bacterial CFU load/ml	−14
	Laroye et al. (25)	2018	*B. Fragilis*	Not significantly decreased bacterial load	UC MSC 10^6 concentration	Bacterial CFU load/ml	−37
	Laroye et al. (25)	2018	*S. aureus*	Not significantly decreased bacterial load	UC MSC 10^6 concentration	Bacterial CFU load/ml	−57
	Laroye et al. (25)	2018	*S. aureus*	Not significantly decreased bacterial load	UC MSC 10^5 concentration	Bacterial CFU load/ml	−66
	Laroye et al. (25)	2018	*S. aureus*	Not significantly decreased bacterial load	UC MSC 10^3 concentration	Bacterial CFU load/ml	−87
10	Chow et al. (28)	2020	*E. coli*	Significantly decreased bacterial load	CM from BM MSC	Bacterial load CFU log10	37
	Chow et al. (28)	2020	*MRSA*	Significantly decreased bacterial load	CM from BM MSC	Bacterial load CFU log10	33
11	Kim et al.(13)	2022	*E. coli*	Significantly reduced bacterial load	UC MSC	Bacterial load CFU/ml	34
	Kim et al.(13)	2022	*E. coli*	Not significantly reduced bacterial load	EVs derived from UC MSC	Bacterial load CFU/ml	14
12	Monsarrat et al. (6)	2019	*Human bacterial subgingival sample*	Significant reduced bacterial load	AT MSC	Bacterial number of CFUs Log	12
	Monsarrat et al. (6)	2019	*E. coli*	Significant reduced bacterial load	AT MSC	Bacterial number of CFUs log	4
	Monsarrat et al. (6)	2019	*L. casei*	Significantly reduced bacterial load	AT MSC	Bacterial number of CFUs log	4
	Monsarrat et al. (6)	2019	*S. sanguinis*	Significantly reduced bacterial load	AT MSC	Bacterial number of CFUs log	3
	Monsarrat et al. (6)	2019	*S. aureus*	Significantly reduced bacterial load	AT MSC	Bacterial number of CFUs log	1
	Monsarrat et al. (6)	2019	*P. gingivalis*	Not significantly reduced bacterial load	AT MSC	Bacterial number of CFUs log	1
	Monsarrat et al. (6)	2019	*F. nucleatum*	Not significantly reducedbacterial load	AT MSC	Bacterial number of CFUs log	0
	Monsarrat et al. (6)	2019	*P. intermedia*	Not significantly reducedbacterial load	AT MSC	Bacterial number of CFUs log	−1
	Monsarrat et al. (6)	2019	*E. faecalis*	Not significantly reduced	AT MSC	Bacterial number of CFUs log	−2

Table shows all studies in which bacterial load was described in terms of CFU count. For each study, the following variables are shown: bacterial strain used, the specific factor derived from MSCs that was utilized, the parameter measured to quantify the bacterial load, and the percentage of reduction after treatment with MSCs or their factors in an *in vitro* setting. %, percentage of reduction of bacterial load; CFU, colony-forming units; BM, bone marrow; EVs extracellular vesicles; AT, adipose tissue; UC, umbilical cord; DP, dental pulp; CM, conditioned medium.

According to the treatment with MSC derivatives or MSC alone as treatment, it was found that the greatest reduction in the percentage of bacterial count was observed in the study utilizing conditioned medium from MSC derived from BM (Lee et al., 2013; Table 2). In their study, MSC were first subjected to interactions with bacteria in an *in vivo* model. Afterwards, the fluid resulting from this interaction was collected and re-exposed to bacteria *in vitro*. The results indicated that pre- conditioning of the medium *in vivo* led to a significant reduction in the second interaction with bacteria, achieving a 97% reduction in bacterial load (Lee et al., 2013; Table 2). Furthermore, studies that primarily used conditioned media produced from AT-, BM-, UC-, and DP-derived MSC reported a percentage reduction in bacterial load above 50% (Figure 3 and Table 2). In four studies (Ren et al., 2020; Yagi et al., 2020; Krasnodembskaya et al., 2010; Sung et al., 2016; Table 2), the utilization of MSC as a standalone treatment yielded a > 50% reduction in bacterial load. A single study used EVs *in vitro*, which resulted in no statistically significant reduction in bacterial growth (Kim et al., 2022; Table 2). The studies selected in this systematic review showed that the antibacterial capacity of a conditioned medium, also known as MSC secretome, would be superior to that of MSC alone.

The types of tissue from which MSC were obtained were also evaluated to describe the differences in outcomes, and it was found that the majority of studies reporting a reduction greater than 60% were those utilizing BM-derived MSC and their CM. Among the articles related to BM MSC, bacterial proliferation was not reported in any study, indicating that this tissue was associated with more favorable outcomes than the others (Figure 3 and Table 2). A < 20% reduction in bacterial count was predominantly reported in studies employing unstimulated CM from UC-derived MSC (Ren et al., 2020; Sung et al., 2016; Figure 3 and Table 2). In addition, one study employing CM from UC-derived MSC showed a 0% effect on bacterial growth, indicating that the therapy did not result in any discernible increase or reduction (Ren et al., 2020; Table 2). Three studies employed *in vitro* AT MSC; among them, most reported favorable outcomes in terms of bacterial reduction (Ren et al., 2020; Yagi et al., 2020; Table 2). Nevertheless, one study exhibited variable results, demonstrating effectiveness in reducing bacteria such as native human subgingival bacteria, *E. coli, L. casei*, and *S. aureus*, but proved ineffective in reducing *F. nucleatum, P. intermedia* and *E. faecalis*. In the case of this last two bacterial strains, increase in bacterial proliferation that was not statistically significant was observed after AT-MSC treatment (Monsarrat et al., 2019; Table 2). A variability was observed on the results using MSC and their derivates from different tissue sources. This suggests that the types of tissue from which MSC were obtained could be a factor determining the effectiveness of bacterial reduction against specific types of bacteria, given that MSC derived from different origins have a range of biological characteristics.

As previously stated, the bacteria that were most commonly used in these studies were *E. coli* and *S. aureus* (Figure 2A). When examining the effect of MSC against these two bacteria in the *in vitro* studies, it was found that the study that used CM derived from AT MSC achieved the highest percentage (78%) of decreased *S. aureus* counts (Wood et al., 2018; Supplementary Figure 2.1 and Table 2). It is also important to highlight that among the studies that used *S. aureus* as a bacterial agent, only one reported an increase in bacterial count after treatment in comparison to the control. In this study, MSC derived from UC were used. This type of MSC had no inhibitory effect on bacterial count (Laroye et al., 2018; Supplementary Figure 2.1 and Table 2). In the case of *E. coli*, it was found that the study that used CM from BM MSC resulted in the greatest percentage (97%) reduction of *E. coli* counts (Lee et al., 2013; Supplementary Figure 2.2 and Table 2). The remaining results demonstrated a wide range of effects, reporting both reductions and non- reductions in bacterial count. No studies have reported an increase in *E. coli* after the use of MSC *in vitro* (Supplementary Figure 2.1).

### 3.2 *In vivo* effects of MSC treatment against bacteria

**FIGURE 4:**
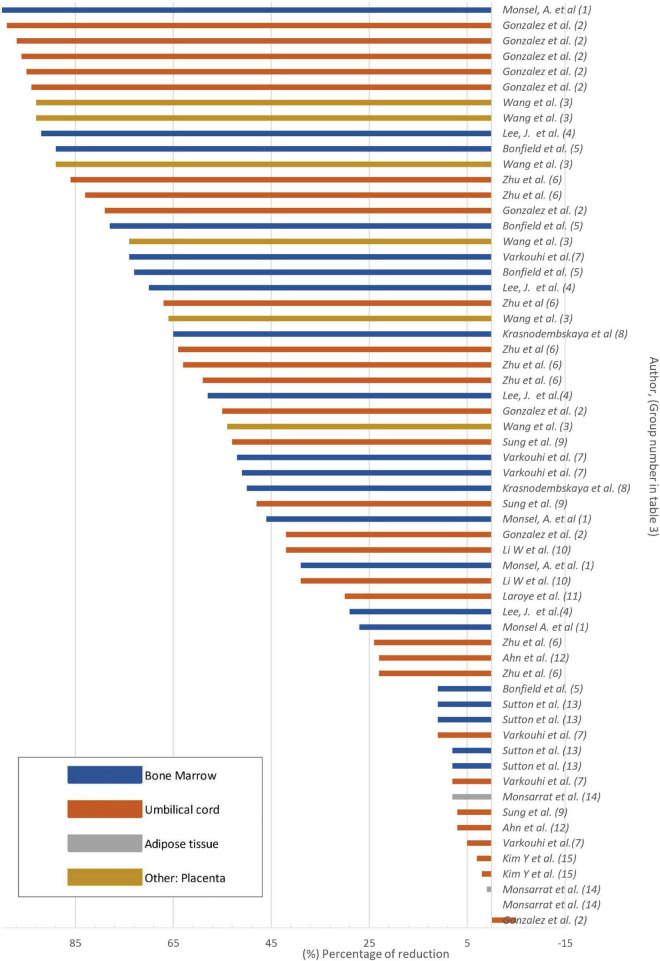
Bacterial load reduction *in vivo* Bar plot. Percentage of bacterial load reduction after treatment with MSCs or MSC-derivatives *in vivo*. Horizontal bar plot showing the percentage reduction after treatment in comparison to control.

Fifteen *in vivo* studies were identified among the selected studies. These studies show a greater number of positive results than the *in vitro* studies (Table 3 and Figure 4). The majority of studies demonstrated a significant decrease in bacterial count in animal models, with one study reporting a 100% bacterial reduction. In this study, treatment with MVs derived from BM-MSC resulted in the inhibition of bacteremia and suppressed bacterial growth in the blood samples obtained from the mouse model (Monsel et al., 2015; Table 3). Nevertheless, it is crucial to emphasize that a considerable proportion of studies reported experimental results in which a significant decrease was not achieved (Table 3 and Figure 4). A single study reported that MSC therapy failed to induce inhibition but instead resulted in the proliferation of bacteria. This study employed the MSC sub-population CD 362+, which was derived from UC tissue (Gonzalez et al., 2020; Table 3).

**TABLE 3:** Percentage reduction of bacterial load *in vivo*.

Group Number	Author-(Appendix reference)	Year	Bacteria	Antibacterial outcomes	Factor used	Sample	%
1	Monsel et al. (20)	2015	*E. coli*	Microvesicles significantly eliminated bacteria CFU	BM MSCs MVs	Blood bacteria count	100
	Monsel et al. (20)	2015	*E. coli*	Microvesicles significantly inhibited bacteria CFU	BM MSCs MVs	Lung homogenate bacterial count	46
	Monsel et al. (20)	2015	*E. coli*	Microvesicles significantly inhibited bacteria CFU	BM MSCs	BAL bacteria count	39
	Monsel et al. (20)	2015	*E. coli*	Microvesicles significantly Inhibited bacteria CFU	BM MSCs MVs	BAL bacterial count	27
2	Gonzalez et al. (18)	2020	*Escherichia spp*	Bacteria significantly reduced on tissue homogenates	CD362+ UC- MSC	Liver homogenate bacterial count	99
	Gonzalez et al. (18)	2020	*Escherichia spp*	Ameriolation but lesser extent on spleen tissue in comparison to liver Tissue	CD362+ UC- MSC	Spleen homogenate bacterial count	96
	Gonzalez et al. (18)	2020	*Klebsiella spp*	Significant amelioration but lesser extent on spleen tissue in comparison to liver tissue	CD362+ UC- MSC	Spleen homogenate bacterial count	97
	Gonzalez et al. (18)	2020	*Klebsiella spp*	Bacteria significantly reduced on tissue homogenates	CD362+ UC- MSC	Liver homogenate bacterial count	95
	Gonzalez et al. (18)	2020	*Enterococcus spp*	Bacteria significantly reduced on tissue homogenates	CD362+ UC- MSC	Liver homogenate bacterial count	94
	Gonzalez et al. (18)	2020	*Polymicrobial*	MSC decreased bacteria	CD362+ UC- MSC	Liver bacterial gene count	79
	Gonzalez et al. (18)	2020	*Polymicrobial*	MSC reduced bacteria	CD362+ UC- MSC	Serum bacterial gene count	55
	Gonzalez et al. (18)	2020	*Polymicrobial*	MSC reduced bacteria	CD362+ UC- MSC	Peritoneum lavage bacterial gene count	42
	Gonzalez et al. (18)	2020	*Enterococcus Spp*	Amelioration but lesser extent on spleen tissue in comparison to liver Tissue	CD362+ UC-MSC	Spleen homogenate bacterial count	−5
3	Wang et al. (17)	2022	*Klebsiella P. strain serotype 2*	MSC treated group had a significant difference in comparison to control	shLuc P-MSC non-target knockdown	Lung homogenate bacterial counts	93
	Wang et al. (17)	2022	*Klebsiella P. strain serotype 2*	MSC treated group had a significant difference in comparison to control	shLuc P-MSC non-target knockdown	Blood bacterial count	93
	Wang et al. (17)	2022	*Klebsiella P. strain serotype 2*	Decreased but not significantly bacterial count	P-MSC	Blood bacterial count	89
	Wang et al. (17)	2022	*Klebsiella P. strain of serotype 2*	MSC knockdown of ILB had a higher bacterial load in comparison to not knockdown target MSC	P-MSC specific knockdown of IL1B	Lung homogenate bacterial count 48h post infection	74
	Wang et al. (17)	2022	*Klebsiella P. strain serotype 2*	Significantly decreased bacterial count	P-MSC	Lung bacterial count	66
	Wang et al. (17)	2022	*Klebsiella P strain of serotype 2*	MSC knockdown of ILB had a higher bacterial load in comparison to not knockdown target MSC	P-MSC specific knockdown of IL1B	Blood bacterial count	54
4	Lee et al. (5)	2012	*E. coli*	Significantly reduced bacterial Load	BM MSC intrabronchially	Alveolar fluid bacterial count	92
	Lee et al. (5)	2012	*E. coli*	Significantly reduced bacterial load	BM MSC double dose 10 × 10^6 intravenously	Alveolar fluid bacterial count	70
	Lee et al. (5)	2012	*E. coli*	Significantly reduced bacterial load	BM MSC Intravenously	Alveolar fluid bacterial count	29
	Lee et al.(5)	2012	*E. coli*	Mesenchymal stem cell eliminated bacteria CFU in ex vivo model	BM MSC Intrabronchially Ex vivo	Alveolar fluid bacterial count	58
5	Bonfield et al. (7)	2021	*M. intracellulare*	MSC significantly decreased bacteria in comparison to the Cystic Fibrosis infected mice Model	BM MSC embedded with *M. intracellulare*	Lung homogenate bacterial count	89
	Bonfield et al. (7)	2021	*M. avium*	MSC significantly decreased bacteria in comparison to the Cystic Fibrosis infected mice Model	BM MSC embedded with *M. Avium*	Lung homogenate bacterial count	78
	Bonfield et al. (7)	2021	*M. avium*	MSC significantly decreased bacteria in comparison to the Wild type infected mice model	BM MSC embedded with *M. avium*	Lung homogenate bacterial count	73
	Bonfield et al. (7)	2021	*M. intracellulare*	MSC decreased bacteria in comparison to the Wild type infected mice model	BM MSC embedded with *M. intracellulare*	Lung homogenate bacterial count	11
6	Zhu et al. (31)	2017	*E. coli*	Significantly decreased bacterial count	UC-MSC	Spleen bacterial count	86
	Zhu et al. (31)	2017	*E. coli*	Significantly decreased bacterial count	UC-MSC	Brain bacterial count	83
	Zhu et al. (31)	2017	*E. coli*	Significantly decreased bacterial count	Preconditioned CM from UC- MSC	Lung bacterial count	67
	Zhu et al. (31)	2017	*E. coli*	Not significantly decreased bacterial count	Preconditioned CM from UC- MSC	Spleen bacterial count	64
	Zhu et al.(31)	2017	*E. coli*	Significantly decreased bacterial count	Preconditioned CM derived from UC-MSC	Blood bacterial count	63
	Zhu et al.(31)	2017	*E. coli*	Significantly decreased bacterial count	UC-MSC	Blood bacterial count	59
	Zhu et al.(31)	2017	*E. coli*	Significantly decreased bacterial count	Preconditioned CM from UC- MSC	Brain bacterial count	24
	Zhu et al.(31)	2017	*E. coli*	Significantly decreased bacteria count	UC-MSC	Lung bacterial count	23
7	Varkouhi et al. (30)	2021	*Polymicrobial*	MSCs significantly decreased bacterial count	BM MSC	Blood bacterial count	74
	Varkouhi et al. (30)	2021	*Polymicrobial*	MSCs significantly decreased bacterial count	BM MSC	Spleen bacterial count	52
	Varkouhi et al. (30)	2021	*Polymicrobial*	MSC significantly decreased the bacterial growth	BM MSC	Lung homogenate bacterial count	51
	Varkouhi et al. (30)	2021	*Polymicrobial*	MSC didn’t affect the bacterial growth	UC MSC	Blood bacterial count	11
	Varkouhi et al. (30)	2021	*Polymicrobial*	MSCs didn’t affect bacterial count	UC MSC	Spleen bacterial count	8
	Varkouhi et al. (30)	2021	*Polymicrobial*	MSC didn’t affect bacterial growth	UC MSC	Lung homogenate bacterial count	5
8	Krasnodembskaya et al. (2)	2010	*E. coli*	MSC significantly inhibited bacterial count	BM MSC	Lung homogenate bacterial count	65
	Krasnodembskaya et al. (2)	2010	*E. coli*	MSC significantly inhibited bacterial count	BM MSC	BAL bacterial count	50
9	Sung et al. (3)	2015	*E. coli*	MSC inhibited bacterial count	UC MSC	BAL bacterial count	53
	Sung et al. (3)	2015	*E. coli*	MSC scrambled with siRNA inhibited bacterial CFU	UC MSC scramble siRNA	BAL bacterial count	48
	Sung et al. (3)	2015	*E. coli*	Antibacterial effect of MSC with knockdown of TLR-4 siRNA was abolished	UC MSC TLR-4 siRNA	BAL bacterial count	7
10	Li et al.(29)	2020	*H. influenzae*	MSC strongly inhibited bacterial growth	UC MSC	BAL bacterial count	42
	Li et al.(29)	2020	*H. influenzae*	MSC strongly inhibited bacterial growth	UC MSC	Lung homogenates bacterial count	39
11	Laroye et al. (25)	2018	*Polymicrobial*	Did not significantly decreased bacterial count	UC MSC	Blood bacterial count	30
12	Ahn et al. (14)	2018	*E. coli*	MSC significantly decreased bacterial count	UC MSC	CSF bacterial count at 24hrs post infection	23
	Ahn et al. (14)	2018	*E. coli*	MSC did not significantly decreased bacteria	UC MSC	CSF bacterial count at 6hrspost infection	7
13	Sutton et al. (24)	2017	*S. aureus*	MSC significantly decreased bacterial count	BM MSC	BAL bacterial count in Wild Type mice model	11
	Sutton et al. (24)	2017	*S. aureus*	MSC significantly decreased bacterial count	BM MSC	BAL bacterial count in Cystic fibrosis mice Model	11
	Sutton et al. (24)	2017	*Pseudomonas aeruginosa*	MSC significantly decreased bacterial count	BM MSC	BAL bacterial count in Wild type mice model	8
	Sutton et al. (24)	2017	*Pseudomonas aeruginosa*	MSC significantly decreased bacterial count	BM MSC	BAL bacterial count in Cystic fibrosis mice Model	8
14	Monsarrat et al. (6)	2019	*Wild periopathogenic bacteria*	MSCs significantly decreased bacterial count	AT MSC graft after 6 weeks	Subgingival sample	8
	Monsarrat et al.(6)	2019	*Wild periopathogenic bacteria*	MSCs minimally reduced bacterial count	AT MSC graft baseline	Subgingival sample	1
	Monsarrat et al.(6)	2019	*Wild periopathogenic bacteria*	MSCs minimally reduced bacterial count	AT MSC graft after 1 week	Subgingival sample	0
15	Kim Y, *et al.* (13)	2022	*E. coli*	MSC did not significantly alter bacterial count	UC MSC EVs	CSF bacterial count	3
		2022	*E. coli*	MSC did not significantly alter bacterial count	UC MSC EVs	CSF bacterial count	2

Results showing the percentage reduction of bacterial load in the collected fluid/tissue samples from *in vivo* models. Table shows the type of bacteria employed, the antibacterial outcomes, the sample used to measure the bacterial load, and the percentage of reduction following MSC treatment or their factors. %, percentage of bacterial load reduction; BAL, bronchoalveolar lavage; siRNA, short interfering RNA; CSF, Cerebrospinal fluid; CM, conditioned medium; shLuc-PMSC, non-target knockdown MSC; P-MSC, placenta-derived MSC; BM, bone marrow; AT, adipose tissue; UC, umbilical cord.

The majority of studies used lung to determine bacterial count. The remaining studies used blood, spleen, bronchoalveolar lavage (BAL), and cerebrospinal fluid (CSF). Only three studies employed liver, peritoneal lavage, serum, brain, and subgingival samples (Gonzalez et al., 2020; Zhu et al., 2017; Monsarrat et al., 2019; Table 3).

Seven *in vivo* studies reported decreased bacterial count in collected samples (Table 3). Six studies (Table 3) reported both reduction and non-reduction after MSC treatment: Gonzales et al. reported a significant decrease in bacterial load in liver homogenates after infection; however, this significant reduction was not seen in the spleen when using CD362+ UC-MSC (Table 3). Wang et al. (Table 3), reported that the use of P-MSC results in a decrease in the number of bacteria in the lungs and blood. However, when IL1B knockdown P-MSC were used, there was no reduction in bacteria in the same samples. This suggests that the expression of IL1B is required for the antibacterial action. Zhu et al. reported that the use of UC MSC led to a reduction in bacterial count in the spleen, brain, and lungs, while the use of CM from UC MSC reduced bacterial count only in the brain and lungs (Table 3). Similarly, Varkouhi et al. reported a reduction of bacterial counts in the blood, spleen, and lungs with the use of BM-MSC but not UC-MSC (Table 3). In addition, Ahn et al. reported that there was no effect from the use of UC-MSC at 6 h post-infection. The only reduction observed was in cerebrospinal fluid at 24 h (Table 3). Similarly, Monsarrat et al. reported that the use of AT MSC only resulted in a decrease in bacterial count at 6 weeks post-treatment (Table 3).

In two studies, the application of MSC failed to result in a reduction in bacterial count in the collected samples. UC MSC and UC MSC-derived EVs were employed in these studies; the analyses were conducted on blood and CSF, respectively. Neither study showed a decrease in bacterial count (Laroye et al., 2018; Kim et al., 2022; Table 3).

Additionally, it was observed that, in contrast to *in vitro* studies, a wider range of MSC from various subpopulations and tissues were employed *in vivo*. Gonzales et al. (Table 3) utilized CD362+ MSC in their investigation, while Wang et al. (Table 3) employed MSC with IL1B knockdown. Additionally, Sung et al. (Table 3) utilized MSC with TLR-4 siRNA knockdown.

It is noteworthy that across the articles examined, a single study included MSC obtained from the placenta, reporting a reduction in bacterial load after treatment (Wang et al., 2023; Table 3). Only one study used EVs as a treatment for cerebrospinal fluid infection and found no significant results of bacterial load reduction (Kim et al., 2022; Table 3). The MSC derived from UC, BM, and P-MSC achieved a > 50% reduction in bacterial load (Table 3 and Figure 4). In contrast, AT-MSC, when employed as a treatment *in vivo*, were reported to reduce the bacterial load by < 10% in comparison to control (Monsarrat et al., 2019; Table 3 and Figure 4).

Among the studies on *E. coli*, it was found that MSC derived solely from BM and UC tissue were used to test their antibacterial effect against *E. coli*, reporting results with a reduction in bacterial count of more than 20% in most studies (Supplementary Figure 2.3 and Table 3). Among the studies on *S. aureus*, a single study used these bacteria *in vivo* (Sutton et al., 2017; Table 3). In this study, a significant reduction of 11% in the bacterial population was observed. The animal models used were cystic fibrosis transmembrane receptor-deficient mice, and congenic background mice were used as controls. Following treatment with BM-MSC, both groups showed a decrease in bacterial count in BAL collected from the animal models (Sutton et al., 2017; Table 3).

It is important to note that two studies employed an *ex vivo* model utilizing human lung tissue (Lee et al., 2013; Park et al., 2019; Table 1), while only one study utilized pig (Laroye et al., 2018; Table 1) and only one study used sheep (Asmussen et al., 2014; Table 1) animal models, with the remaining studies employing murine models, which was the most commonly utilized animal model (Table 1). Thus, the use of large animal models to investigate the application of MSC has infrequently been reported. Infection of different tissues showed variations in the results among the selected studies. The antibacterial inhibitory effect may be correlated with either the type or subpopulation of MSC used and the type of infected tissue.

## 4 Discussion

MSC have been reported to have direct antibacterial properties by secreting peptides that disrupt several elements of the bacterial structure, including the plasma membrane (Zhang et al., 2021). The findings from the studies included in this systematic review indicate that MSC and their derivatives, originating from various tissues, exhibit diverse potentials for bacterial reduction (Table 1). This systematic review focused on describing variations in the use of MSC or their components derived from different tissues and evaluated their effects on various bacterial strains and animal models. In the present systematic review, it was observed that a majority of the studies yielded positive outcomes in terms of reducing bacterial counts (Table 1). Upon comparing the effects observed *in vitro* and *in vivo*, it was confirmed that more *in vivo* studies reported a > 50% reduction in the bacterial load in

comparison to *in vitro* studies. This suggests that the bacterial reduction capacity with MSC treatment could also work under complex *in vivo* biological conditions that assemble human physiology (Table 3 and Figure 4).

Among the articles reviewed, it was observed that the use of BM-derived MVs *in vivo* resulted in a decrease in the total bacterial count (100%) (Monsel et al., 2015; Table 3). This suggests that MSC secrete products that can be considered as viable options for cell-free therapy. It is important to highlight that treatment with MSC or their derivatives has shown a broad spectrum of bacterial reduction capacity after treatment, and a significant decrease in bacterial load was reported in studies using Gram-positive, Gram-negative bacteria, and even antibiotic-resistant bacterial strains (Table 2), suggesting that the use of MSC or their derivatives may provide broad coverage for pathogens.

Our systematic review confirmed the notable antimicrobial activity of MSC, enabling the description of variations in terms of bacterial reduction in the use of different tissues and factors derived from MSC. Our data will guide future research to select the appropriate cell type for different bacterial strains. Additionally, MSC can be considered a therapeutic option because of their *in vivo*-proven antibacterial potential. This result paves the way for further research in larger animal models and biological systems that closely resemble humans, ultimately leading to translational research.

### 4.1 Differences among antimicrobial effects were found in MSC derived from various tissues

**FIGURE 5:**
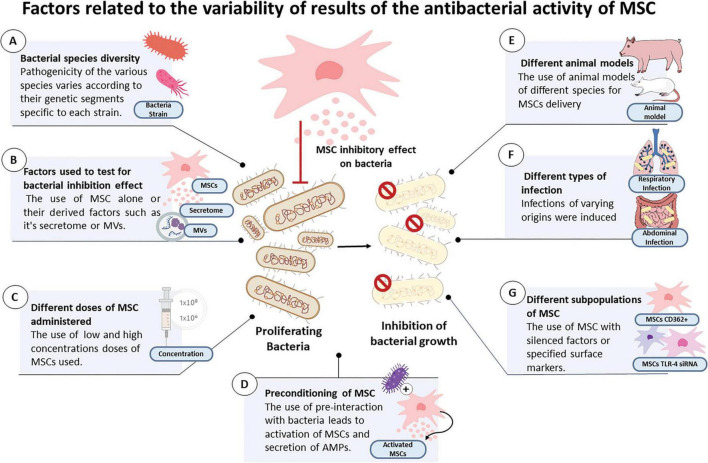
Figure illustrating the main factors linked to the variability of results of the inhibitory effects of MSC or their derivatives on the reported bacteria among the selected articles, which include: **(A)** bacterial species diversity, **(B)** factors used to test for bacterial inhibition effect, **(C)** different doses of MSC administered, **(D)** preconditioning of MSC, **(E)** different animal models, **(F)** different types of infection and **(G)** different subpopulations of MSC.

Among the *in vitro* experimental design studies, the highest bacterial count reduction (97%) was observed in one study in which BM- derived MSC were used (Lee et al., 2013; Table 2). Similarly, among the *in vivo* reports, the highest percentage of bacterial load reduction (100%) was found in the group treated with BM MSC-derived MVs (Monsel et al., 2015; Table 3). Both used *E. coli* to evaluate antibacterial potency. This consistent outcome reflects that the use of BM as a tissue has an antimicrobial effect against certain pathogens. In the remaining studies, both *in vivo* and *in vitro* models exhibited high and low percentages of findings within each study, respectively (Figures 3, 4 and Tables 2, 3). The differences observed among the various studies can be attributed to the following factors:

Variations in the pathogenicity of each bacterium are dictated by virulence factors that differ among microorganisms (Fernández et al., 2004). The virulence factors of bacteria such as toxin presentation, are determined by genetic segments known as pathogenic islands. These pathogenicity islands (PAIs) vary from one bacterium to another (Chatterjee and Raval, 2019); therefore, genome differences may determine the variations in bacterial reduction after MSC treatment between different strains, as some may have a higher colonization capacity, which is dependent on their PAIs (Figure 5A).

The component-MVs, conditioned medium, supernatant or MSC that were utilized: MSC alone act by excreting active molecules with the disadvantage of not having the ability to adhere to a target for a long period, especially in conditions of inflammation (Lombardo et al., 2015), which may determine differences in the efficacy of MSC when used *in vivo* models where infection is accompanied by a local inflammatory process. Previous studies have substantiated this finding, reporting that successful transplantation of MSC is not always viable (Eleuteri and Fierabracci, 2019). CMs and MVs derived from MSC have the advantage of being able to interact and transfer cargo, such as miRNAs (Sandonà et al., 2021), which gives them an additional advantage in terms of effective administration. Among the selected studies, both *in vivo* and *in vitro* cases described the use of CM demonstrating greater efficacy in terms of bacterial reduction in comparison to the use of MSC alone, while MVs showed a strong bacterial growth inhibiting effect (Tables 2, 3 and Figure 5B).

The administered dose of MSC: Previous studies have reported that different dosages and administration timings of the dose are factors associated with increased survival in animal models of sepsis (Lalu et al., 2016). The use of high doses of MSC has been previously reported to enable survival in infected animal models, indicating a correlation between dosage and favorable outcomes (Li et al., 2023). Our review of studies also found that the use of double and high doses produced a significant decrease in bacteria in two *in vivo* models (Horie et al., 2020; Devaney et al., 2015; Table 1). This suggests that a factor that may determine the reduction in bacterial count is the high or low concentration of the doses used. Hence, future research should aim to establish the optimal dosage for different types of infections, in order to determine the effective antibacterial dose of MSC (Figure 5C).

Preconditioning of MSC or their components before treatment: Previous studies have suggested that interactions with bacterial stimuli may lead to MSC activation. It has been reported that after stimulation, there is an increase in antimicrobial peptides secreted by MSC (Yang et al., 2022), which is a preconditioning method that increases antibacterial effects (Yang et al., 2022). This could determine the differences in the reduction of bacterial load in activated MSC versus the use of MSC without previous stimulation. In our systematic review, there were numerous articles in which MSC or their CM before treatment were challenged with bacterial interactions (Table 2). This condition led to positive results in terms of a significant decrease in bacterial count (Figure 5D).

Additionally, *in vivo* studies have shown variability in results possibly due to variability in the animal models that they employed [e.g., rats, mice, sheep, and pigs (Figure 5E)]; the infection that they models tested; the foci of origin that were targeted [e.g., respiratory, abdominal, or sepsis-related infections (Figure 5F)]; and the subpopulations of MSC that were used (e.g., CD362+; MSC with knockdown of IL1B, MSC with TLR-3 agonist, and MSC with knockdown of TLR-4 siRNA) (Table 1 and Figure 5G).

Furthermore, it is important to highlight that the present systematic review included MSC derived from various human tissues, being this a factor that contributed to the variability since the biological properties of MSC vary depending on their tissue of origin. The environment in which a MSC grow is influenced by heterogeneous cellular communities, endowing them with diverse properties (Costa et al., 2021). Consequently, the antibacterial properties of MSCs can vary according to the tissue source from which they are derived.

### 4.2 Limited number of reports assessing the antimicrobial effect of EVs or MVs

This systematic review identified the utilization of EVs and MVs in a total of three studies, with one study applying them *in vitro* (Kim et al., 2022; Table 2) and the other two applying them in an *in vivo* model (Monsel et al., 2015; Kim et al., 2022; Table 3). The limited research conducted on the impact of EVs indicates that there is a shortage of research into the potential of this stem cell-derived component. Previous studies have established that EVs released by MSC play a role in modulating the inflammatory response by facilitating the transfer of mRNA, microRNAs, and proteins (Chen et al., 2016). However, few studies have addressed whether these EVs have the potential to augment peptide secretion via activation of signaling factors (Alcayaga-Miranda et al., 2017).

### 4.3 Lack of standardization

The variation in experimental conditions involving MSC or their constituents plays an important role in yielding favorable outcomes *in vivo* and *in vitro* studies. MSC-derived CM was the predominant component utilized *in vitro* (Table 2). The absence of standardization of the cell growth settings could have led to variations in the results obtained. Prior research has emphasized the significance and requirement of standardizing the manufacturing process of MSC, specifically in relation to the culture media and supplements used, length of culture, and specific conditions under which the culture takes place (Vizoso et al., 2017).

### 4.4 Limitations of the study

The main aim of this study was to conduct an extensive literature review on the inherent antibacterial capabilities of MSC or their derivatives, with the aim of evaluating the efficacy of MSC therapy across various tissues. Hence, the present study excluded research that examined the combined administration of MSC or their constituents with other treatments (e.g., immune cells, cytokines, or antibiotics). However, investigating the potential synergistic effects of cell therapy with antibiotics or other components remain an important aspect that should be explored in future studies.

The use of human donor-derived MSC was one of the criteria considered during the selection of studies for inclusion. Previous studies have established that MSC generally lack immunogenic properties, thereby enabling their transplantation into allogeneic hosts without immunosuppression (Musiał-Wysocka et al., 2019). However, existing research has not thoroughly investigated the donor characteristics that could affect the antibacterial properties of MSC, such as sex and age. A previous study have indicated that MSC functions, specifically BM derived MSC proliferation, reduced in relation to aging and in females donors, (Selle et al., 2022) yet it is necessary to determine whether other functions, such as the production of soluble derivatives as antimicrobial peptides, may also reduce their efficacy against bacteria in different donor age and sex. These aspects require further assessment and may potentially influence the observed variations in the effects exerted by MSC.

In addition, determining the factors associated with the antibacterial mechanism of MSCs is an important step to optimize the effects of these cells. Only twelve studies showed potential candidates involved in the bacterial growth inhibitory mechanism. The majority of the studies identified LL-37 as an antibacterial mediator released by MSCs (Table 1). This finding was consistent with previous research indicating that the production of antimicrobial peptides plays a role in directly inhibiting bacterial growth (Alcayaga-Miranda et al., 2017). Although most studies described an inhibitory effect on bacteria, further research is required to explore the factors associated with the antibacterial mode of action of MSC in order to identify the specific elements that are present and are relevant.

## 5 Conclusion

Our results support the antibacterial capabilities of MSC, as most previous studies have reported favorable outcomes in terms of bacterial load decrease. A considerable efficacy rate was observed, particularly when MSC obtained from BM were used. Furthermore, the majority of studies achieved a substantial decrease in the presence of both gram-positive and gram-negative bacteria, demonstrating their broad spectrum of action. However, it is important to note that the antibacterial outcomes varied across different tissues. The response of various bacteria to MSC, the factors employed, cell preconditioning, and concentration are some of the factors that resulted in a wide range of variability in the findings within each of the studies included in the present systematic review. The investigation of the antibacterial effects of MSC is a developing area of study that has received significant attention in the past decade. The field of study in this area continues to grow, providing therapeutic possibilities for the utilization of MSC in the treatment of infectious diseases. While our findings highlight the potential antibacterial effects of MSC and their derivatives, further research is needed to elucidate the factors that determine the effectiveness of their antimicrobial activity and their various components.

## Data Availability

The datasets used in this systematic are available from the corresponding author on request.

## Footnotes

1
https://apps.automeris.io/wpd/
