# Supplementary material for: Antibacterial effects of human mesenchymal stem cells and their derivatives: a systematic review
Source: Front Microbiol. 2024 Sep 25;15:1430650. doi: 10.3389/fmicb.2024.1430650 (PMC11461301; doi:10.3389/fmicb.2024.1430650)
Supplement: Supplementary file 1 [file Data_Sheet_1.PDF]

Supplementary Data 1. Appendix -List of articles included for the analysis [1–31].

1. Bahroudi, M., Bakhshi, B., Soudi, S., Najar-Peerayeh, S. (2020). Antibacterial and antibiofilm activity of bone marrow-derived human mesenchymal stem cells secretome against Vibrio cholerae. Microb Pathog, 139, 103867. https://doi.org/10.1016/j.micpath.2019.103867
2. Krasnodembskaya, A., Song, Y., Fang, X., Gupta, N., Serikov, V., Lee, J.-W., Matthay, M.A., (2010). Antibacterial effect of human mesenchymal stem cells is mediated in part from secretion of the antimicrobial peptide LL-37. Stem Cells, 28, 2229–2238. https://doi.org/10.1002/stem.544
3. Sung, D.K., Chang, Y.S., Sung, S.I., Yoo, H.S., Ahn, S.Y., Park, W.S. (2016). Antibacterial effect of mesenchymal stem cells against Escherichia coli is mediated by secretion of beta-defensin- 2 via toll- like receptor 4 signalling. Cell Microbiol, 18, 424–436. https://doi.org/10.1111/cmi.12522
4. Yagi, H., Chen, A.F., Hirsch, D., Rothenberg, A.C., Tan, J., Alexander, P.G., Tuan, R.S. (2020). Antimicrobial activity of mesenchymal stem cells against Staphylococcus aureus. Stem Cell Res Ther, 11, 293. https://doi.org/10.1186/s13287-020-01807-3
5. Lee, J.W., Krasnodembskaya, A., McKenna, D.H., Song, Y., Abbott, J., Matthay, M.A. (2013). Therapeutic effects of human mesenchymal stem cells in ex vivo human lungs injured with live bacteria. Am J Respir Crit Care Med, 187, 751–760. https://doi.org/10.1164/rccm.201206-0990OC
6. Monsarrat, P., Kémoun, P., Casteilla, L., Planat-Bénard, V. (2019). Broad-Spectrum Antibacterial Effects of Human Adipose-Derived Stromal Cells. Stem Cells Int, 2019, 5389629. https://doi.org/10.1155/2019/5389629
7. Bonfield, T.L., Sutton, M.T., Fletcher, D.R., Folz, M.A., Ragavapuram, V., Somoza, R.A., Caplan, A.I. (2021). Donor-defined mesenchymal stem cell antimicrobial potency against nontuberculous mycobacterium. Stem Cells Transl Med, 10(8), 1202–1216. https://doi.org/10.1002/sctm.20-0521
8. Perlee, D., de Vos, A.F., Scicluna, B.P., Mancheño, P., de la Rosa, O., Dalemans, W., Nürnberg, P., Lombardo, E., van der Poll, T. (2019). Human Adipose-Derived Mesenchymal Stem Cells Modify Lung Immunity and Improve Antibacterial Defense in Pneumosepsis Caused by Klebsiella pneumoniae. Stem Cells Transl Med, 8(8), 785–796. https://doi.org/10.1002/sctm.18-0260
9. Wood, C.R., Al Dhahri, D., Al Delfi, I., Pickles, N.A., Sammons, R.L., Worthington, T., Wright, K.T., Johnson, W.E.B. (2018). Human adipose tissue-derived mesenchymal stem/stromal cells adhere to and inhibit the growth of Staphylococcus aureus and Pseudomonas aeruginosa. J Med Microbiol, 67, 1789–1795. https://doi.org/10.1099/jmm.0.000861
10. Ren, Z., Zheng, X., Yang, H., Zhang, Q., Liu, X., Zhang, X., Yang, S., Xu, F., Yang, J. (2020). Human umbilical-cord mesenchymal stem cells inhibit bacterial growth and alleviate antibiotic resistance in neonatal imipenem-resistant Pseudomonas aeruginosa infection. Innate Immun, 26, 215–221. https://doi.org/10.1177/1753425919883932
11. Yang, H., Xu, F., Zheng, X., Yang, S., Ren, Z., Yang, J. (2022). Human Umbilical Cord Mesenchymal Stem Cells Prevent Bacterial Biofilm Formation. Biomed Res Int, 2022, 1530525. https://doi.org/10.1155/2022/1530525
12. Dubus, M., Varin, J., Papa, S., Rammal, H., Chevrier, J., Maisonneuve, E., Mauprivez, C., Mongaret, C., Gangloff, S.C., Reffuveille, F., Kerdjoudj, H. (2020). Interaction of

Cutibacterium acnes with human bone marrow derived mesenchymal stem cells: a step toward understanding bone implant- associated infection development. Acta Biomater, 104, 124–134. https://doi.org/10.1016/j.actbio.2019.12.028

13. Kim, Y.-E., Ahn, S.-Y., Park, W.-S., Sung, D.-K., Sung, S.-I., Yang, M.-S., Chang, Y.-S. (2022). Mesenchymal-Stem-Cell-Derived Extracellular Vesicles Attenuate Brain Injury in Escherichia coli Meningitis in Newborn Rats. Life, 12(7), 1030. https://doi.org/10.3390/life12071030
14. Ahn, S.Y., Chang, Y.S., Kim, Y.E., Sung, S.I., Sung, D.K., Park, W.S. (2018). Mesenchymal stem cells transplantation attenuates brain injury and enhances bacterial clearance in Escherichia coli meningitis in newborn rats. Pediatr Res, 84(5), 778–785. https://doi.org/10.1038/s41390-018-0142-5
15. McCarthy, S.D., Horgan, E., Ali, A., Masterson, C., Laffey, J.G., MacLoughlin, R., O'Toole, D. (2020). Nebulized Mesenchymal Stem Cell Derived Conditioned Medium Retains Antibacterial Properties Against Clinical Pathogen Isolates. J Aerosol Med Pulm Drug Deliv, 33, 140–152. https://doi.org/10.1089/jamp.2019.1542
16. Ravenscroft, H., El Karim, I., Krasnodembskaya, A.D., Gilmore, B., About, I., Lundy, F.T., (2022). Novel Antibacterial Properties of the Human Dental Pulp Multipotent Mesenchymal Stromal Cell Secretome. Am J Pathol, 192, 956–969. https://doi.org/10.1016/j.ajpath.2022.02.005
17. Wang, L.-T., Yen, B.L., Wang, H.-H., Chao, Y.-Y., Lee, W., Huang, L.-Y., Chiu, S.-K., Siu, L.K., Liu, K.-J., Sytwu, H.-K., Yen, M.-L. (2023). Placental mesenchymal stem cells boost M2 alveolar over M1 bone marrow macrophages via IL-1β in Klebsiella-mediated acute respiratory distress syndrome. Thorax, 78(5), 504–514. https://doi.org/10.1136/thoraxjnl-2021-217928
18. Gonzalez, H., Keane, C., Masterson, C.H., Horie, S., Elliman, S.J., Higgins, B.D., Scully, M., Laffey, J.G., O'Toole, D. (2020). Umbilical Cord-Derived CD362+ Mesenchymal Stromal Cells Attenuate Polymicrobial Sepsis Induced by Caecal Ligation and Puncture. Int J Mol Sci, 21, 8270. https://doi.org/10.3390/ijms21218270
19. Horie, S., Masterson, C., Brady, J., Loftus, P., Horan, E., O'Flynn, L., Elliman, S., Barry, F., O'Brien, T., Laffey, J.G., O'Toole, D. (2020). Umbilical cord-derived CD362+ mesenchymal stromal cells for E. coli pneumonia: impact of dose regimen, passage, cryopreservation, and antibiotic therapy. Stem Cell Res Ther, 11(1), 116. https://doi.org/10.1186/s13287-020-01624-8
20. Monsel, A., Zhu, Y., Gennai, S., Hao, Q., Hu, S., Rouby, J.-J., Rosenzwajg, M., Matthay, M.A., Lee, J.W. (2015). Therapeutic Effects of Human Mesenchymal Stem Cell-derived Microvesicles in Severe Pneumonia in Mice. Am J Respir Crit Care Med 192, 324–336. https://doi.org/10.1164/rccm.201410-1765OC
21. Krasnodembskaya, A., Samarani, G., Song, Y., Zhuo, H., Su, X., Lee, J.-W., Gupta, N., Petrini, M., Matthay, M.A. (2012). Human mesenchymal stem cells reduce mortality and bacteremia in gram-negative sepsis in mice in part by enhancing the phagocytic activity of blood monocytes. Am J Physiol Lung Cell Mol Physiol, 302, L1003-1013. https://doi.org/10.1152/ajplung.00180.2011
22. Asmussen, S., Ito, H., Traber, D.L., Lee, J.W., Cox, R.A., Hawkins, H.K., McAuley, D.F., McKenna, D.H., Traber, L.D., Zhuo, H., Wilson, J., Herndon, D.N., Prough, D.S., Liu, K.D., Matthay, M.A., Enkhbaatar, P. (2014). Human mesenchymal stem cells reduce the severity of acute lung injury in a sheep model of bacterial pneumonia. Thorax, 69, 819–825. https://doi.org/10.1136/thoraxjnl-2013-204980

23. Devaney, J., Horie, S., Masterson, C., Elliman, S., Barry, F., O'Brien, T., Curley, G.F., O'Toole, D., Laffey, J.G. (2015). Human mesenchymal stromal cells decrease the severity of acute lung injury induced by E. coli in the rat. Thorax, 70, 625–635. https://doi.org/10.1136/thoraxjnl-2015-206813
24. Sutton, M.T., Fletcher, D., Episalla, N., Auster, L., Kaur, S., Gwin, M.C., Folz, M., Velasquez, D., Roy, V., van Heeckeren, R., Lennon, D.P., Caplan, A.I., Bonfield, T.L. (2017). Mesenchymal Stem Cell Soluble Mediators and Cystic Fibrosis. J Stem Cell Res Ther, 7, 400. https://doi.org/10.4172/2157-7633.1000400
25. Laroye, C., Lemarié, J., Boufenzer, A., Labroca, P., Cunat, L., Alauzet, C., Groubatch, F., Cailac, C., Jolly, L., Bensoussan, D., Reppel, L., Gibot, S. (2018). Clinical-grade mesenchymal stem cells derived from umbilical cord improve septic shock in pigs. Intensive Care Med Exp 6, 24. https://doi.org/10.1186/s40635-018-0194-1
26. Masterson, C., Devaney, J., Horie, S., O'Flynn, L., Deedigan, L., Elliman, S., Barry, F., O'Brien, T., O'Toole, D., Laffey, J.G. (2018). Syndecan-2-positive, Bone Marrow-derived Human Mesenchymal Stromal Cells Attenuate Bacterial-induced Acute Lung Injury and Enhance Resolution of Ventilator-induced Lung Injury in Rats. Anesthesiology, 129, 502–516. https://doi.org/10.1097/ALN.0000000000002327
27. Park, J., Kim, S., Lim, H., Liu, A., Hu, S., Lee, J., Zhuo, H., Hao, Q., Matthay, M.A., Lee, J.-W. (2019). Therapeutic effects of human mesenchymal stem cell microvesicles in an ex vivo perfused human lung injured with severe E. coli pneumonia. Thorax 74, 43–50. https://doi.org/10.1136/thoraxjnl-2018-211576
28. Chow, L., Johnson, V., Impastato, R., Coy, J., Strumpf, A., Dow, S. (2020). Antibacterial activity of human mesenchymal stem cells mediated directly by constitutively secreted factors and indirectly by activation of innate immune effector cells. Stem Cells Transl Med, 9, 235–249. https://doi.org/10.1002/sctm.19-0092
29. Li, W., Chen, W., Huang, S., Tang, X., Yao, G., Sun, L. (2020). Mesenchymal Stem Cells Enhance Pulmonary Antimicrobial Immunity and Prevent Following Bacterial Infection. Stem Cells Int 2020, 3169469. https://doi.org/10.1155/2020/3169469
30. Varkouhi, A.K., He, X., Teixeira Monteiro, A.P., Amatullah, H., Tsoporis, J.N., Gupta, S., Ektesabi, A.M., Mei, S.H.J., Stewart, D.J., Keating, A., Dos Santos, C.C. (2021). Immunophenotypic characterization and therapeutics effects of human bone marrow- and umbilical cord-derived mesenchymal stromal cells in an experimental model of sepsis. Exp Cell Res, 399, 112473. https://doi.org/10.1016/j.yexcr.2021.112473
31. Zhu, Y., Xu, L., Collins, J.J.P., Vadivel, A., Cyr-Depauw, C., Zhong, S., Mense, L., Möbius, M.A., Thébaud, B. (2017). Human Umbilical Cord Mesenchymal Stromal Cells Improve Survival and Bacterial Clearance in Neonatal Sepsis in Rats. Stem Cells Dev, 26(14), 1054–1064. https://doi.org/10.1089/scd.2016.0329
