# Supplementary material for: Antibacterial effects of human mesenchymal stem cells and their derivatives: a systematic review
Source: Front Microbiol. 2024 Sep 25;15:1430650. doi: 10.3389/fmicb.2024.1430650 (PMC11461301; doi:10.3389/fmicb.2024.1430650)

Supplement Figure 2.1 Percentage of *S.aureus* load reduction after MSCs or derivatives treatment-*In vitro*. Horizontal bar chart showing percentage of reduction after comparing treatment with control experimental groups.

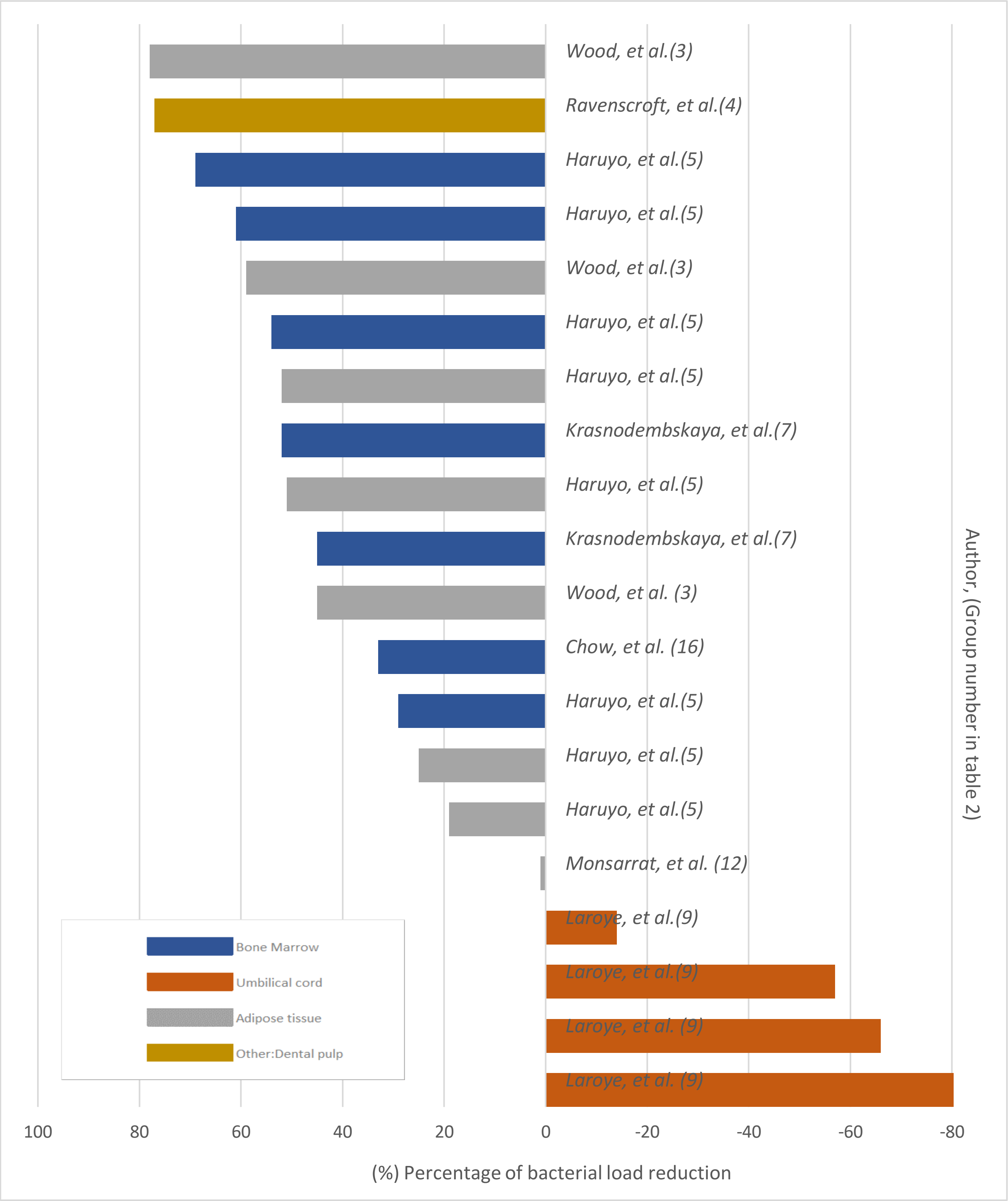

Supplement Figure 2.2 Percentage of *E.coli* load reduction after MSCs or derivatives treatment-*In vitro*. Horizontal bar chart showing percentage of reduction after comparing treatment with control experimental groups

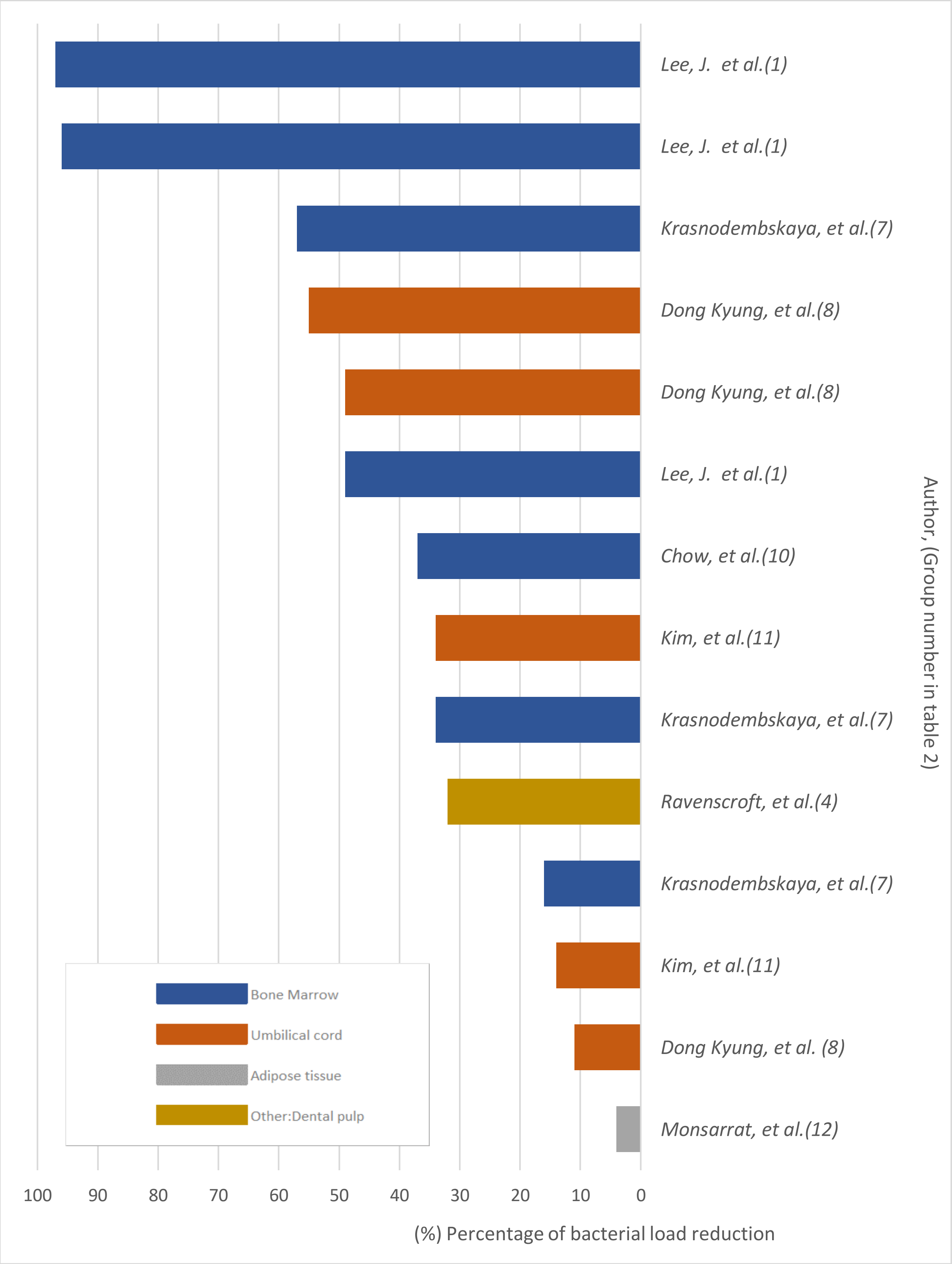

Supplement Figure 2.3 Percentage of *E.coli* load reduction after MSCs or derivatives treatment-*In vivo.* Horizontal bar chart showing percentage of reduction after comparing treatment with control experimental groups.

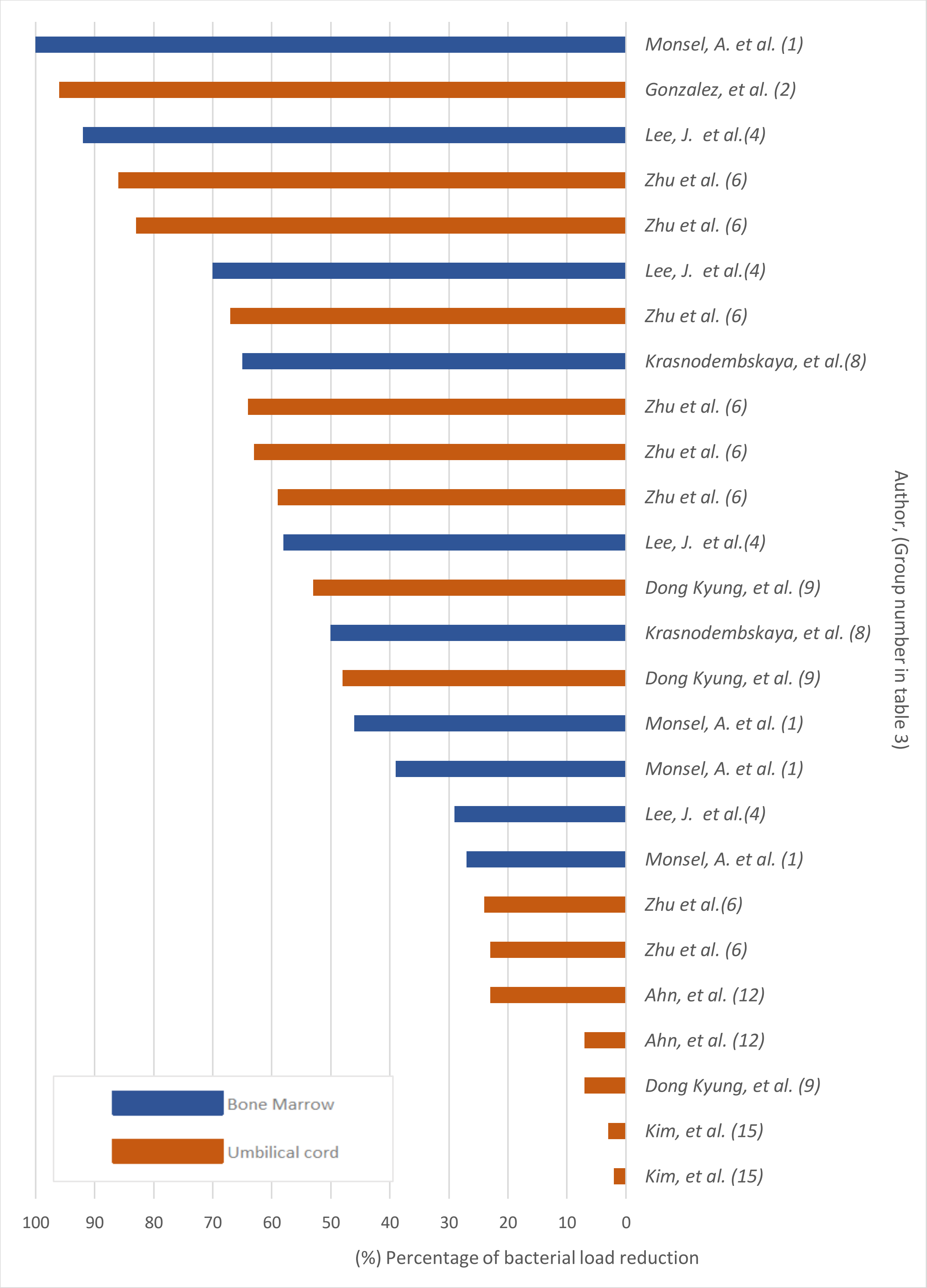

Supplement: Supplementary file 2 [file Data_Sheet_2.PDF]
